# Promise for plant pest control: root-associated pseudomonads with insecticidal activities

**DOI:** 10.3389/fpls.2013.00287

**Published:** 2013-07-31

**Authors:** Peter Kupferschmied, Monika Maurhofer, Christoph Keel

**Affiliations:** ^1^Department of Fundamental Microbiology, University of LausanneLausanne, Switzerland; ^2^Plant Pathology, Institute of Integrative Biology, Swiss Federal Institute of Technology ZurichZurich, Switzerland

**Keywords:** *Pseudomonas*, insecticidal, plant-associated, entomopathogens, toxins, biocontrol, rhizosphere, *Bacillus*

## Abstract

Insects are an important and probably the most challenging pest to control in agriculture, in particular when they feed on belowground parts of plants. The application of synthetic pesticides is problematic owing to side effects on the environment, concerns for public health and the rapid development of resistance. Entomopathogenic bacteria, notably *Bacillus thuringiensis* and *Photorhabdus*/*Xenorhabdus* species, are promising alternatives to chemical insecticides, for they are able to efficiently kill insects and are considered to be environmentally sound and harmless to mammals. However, they have the handicap of showing limited environmental persistence or of depending on a nematode vector for insect infection. Intriguingly, certain strains of plant root-colonizing *Pseudomonas* bacteria display insect pathogenicity and thus could be formulated to extend the present range of bioinsecticides for protection of plants against root-feeding insects. These entomopathogenic pseudomonads belong to a group of plant-beneficial rhizobacteria that have the remarkable ability to suppress soil-borne plant pathogens, promote plant growth, and induce systemic plant defenses. Here we review for the first time the current knowledge about the occurrence and the molecular basis of insecticidal activity in pseudomonads with an emphasis on plant-beneficial and prominent pathogenic species. We discuss how this fascinating *Pseudomonas* trait may be exploited for novel root-based approaches to insect control in an integrated pest management framework.

## INTRODUCTION

With the world population still experiencing continuous growth, an immediate priority of agriculture is to increase crop production to assure food security while becoming more sustainable (Gatehouse et al., 2011). One way to do so is by improving the management of pests. Weeds, plant pathogens, and certain animal species are considered to be the major pests of economic significance and together they are estimated to reduce the world’s annual crop yield by approximately 30–40% (Oerke, 2006). Due to their incredible diversity and adaptability, insects are probably the single most challenging pest to control in agriculture worldwide. Insects do not only cause major damage to agricultural crops as pests, but are also vectors of diseases. Since the introduction of synthetic insecticides, their application has made a major contribution to improve food production, but it was also soon discovered to be problematic in many ways. The rapid appearance of resistance to insecticides is a major concern in pest management. Today insect pest species of economic importance as pests that are resistant to more than 30 different chemical insecticides are no longer a rarity (Arthropod Pesticide Resistance Database, Michigan State University). Moreover, chemical insecticides are troublesome because of their potentially nocuous effects on the environment and public health (Heckel, 2012).

After decades of intensive pesticide application, it has become evident that there is no silver bullet solution to the control of pests in sustainable agriculture. The integration of many different, complementary approaches of chemical and biological control methods to solve the diverse and challenging problems with pests is the basic idea behind integrated pest management (IPM) programs (van den Bosch and Stern, 1962). Since its inception, IPM has become an increasingly important and popular toolbox-like approach to protect plants in agriculture against weeds, pathogens and animal pests (Oerke, 2006). Its tactics are designed to decrease the amount of chemical pesticides applied through careful forecasting or even to replace them by biological alternatives. The two main alternatives to synthetic insecticides are the exploitation of semiochemicals (like pheromones) to change the behavior of insects or the use of biological control agents (parasites, predators, and pathogens) to reduce the pest population size (Bale et al., 2008). IPM-based systems are becoming progressively more popular due to the increased public awareness of the above-mentioned problematic effects of synthetic pesticides and interest in the development of alternative approaches for plant pest control. Microbial products that are based on insecticidal microorganisms for biological pest management strategies are receiving particular attention.

*Bacillus thuringiensis* (Bt) is a Gram-positive, spore-forming soil bacterium and the insecticidal organism which is dominating the market for products for microbial control of insects (Bravo et al., 2011; Sanahuja et al., 2011). The reason for its success is the production of pore-forming δ-endotoxins, namely Cry and Cyt proteins (Crickmore et al., 1998, 2013). These so-called crystal proteins are produced during sporulation and show potent and specific insecticidal activity. Once proteolytically activated, the Cry and Cyt proteins act in the midgut of insects as pore-forming toxins via binding to specific receptors or directly to membrane lipids, respectively (Bravo et al., 2007; Vachon et al., 2012). In addition to the well-known crystal toxins, Bt produces an array of additional virulence factors that contribute to the insecticidal activity of this bacterium (Nielsen-LeRoux et al., 2012). Bt is typically applied as topical sprays and has several advantages over conventional chemical insecticides. The bacterium’s pathogenic activity is specific toward a narrow range of insect species and its application is considered to be environmentally sound and harmless to humans and other mammals. However, the use of Bt as a biological control agent has some limitations. The bacterium shows low environmental persistence after topical application, mainly because it is sensitive to solar irradiation as well as to the chemical environment on plant leaves, and is not a competitive plant colonizer (Bizzarri and Bishop, 2008; Raymond et al., 2010). Therefore, and because the susceptible stages of the pest insects are during the early instar larvae, Bt provides only short-term crop protection in the field and requires precise application practices (Bravo et al., 2011). The recent discovery that at least some Bt strains are capable of colonizing crop plants as endophytes and as such translocate throughout the plant (Monnerat et al., 2009) may open up an avenue for new Bt application strategies.

To overcome the problem of the low persistence of Bt on plants, genetically modified (GM) crops that express variants of the Cry toxins have been developed and successfully commercialized. Planting of GM crops reduced the amount of pesticides applied by 8.9% in the period from 1996 to 2011 (James, 2012) and is a component of IPM strategies due to its compatibility with biological control methods (Bale et al., 2008). However, the major drawback of this new biotechnology has been the development of resistance against the Cry toxins by pests (Bravo et al., 2011). Due to the relatively simple mode of action of Cry toxins and the absence of complementary virulence factors normally found in the complete microorganism, resistance is much more probable to develop toward the insect toxin in the GM plants than to the entire microorganism (Cory and Franklin, 2012). Infections by microbial pathogens are complex and likely to require more diverse polygenic resistance mechanisms in pest insects. In addition, there are public concerns about transgenic crops regarding their impact on biodiversity and the consumer’s health and the possible dependency of farmers on seed companies. Especially in Europe, the public acceptance for GM crops is currently fairly low for these reasons.

Belowground pest insects are especially difficult to control, because they are hidden in the soil and therefore hard to detect and to get access to. Although root herbivory can cause significant damage to crops, even leading to a sudden collapse of the plant population, there is still a considerable lack of research data about root feeders and their impact on plants (Hunter, 2001; Blossey and Hunt-Joshi, 2003). For instance, the Western corn rootworm *Diabrotica virgifera* is a significant economic pest insect of maize in the United States and in Europe and acquired the nickname “billion dollar bug,” not without reason (Gray et al., 2009). Even if this troublesome insect species has been the subject of many scientific studies, this root feeder remains challenging to control because of its cryptic lifestyle, the adaptation to crop rotation, and the development of resistance to certain insecticides. While the use of chemical pesticides for pest management in soils is extremely restricted, microbial control is a promising approach to address problems with soil-dwelling insects due to the more favorable environmental conditions for microbes in contrast to aboveground habitats (e.g., absence of ultraviolet radiation and lower risk of desiccation in the soil). Species of *Photorhabdus* and *Xenorhabdus*, bacteria which are living in symbiosis with entomopathogenic nematodes, are used in agriculture as soil-applied insecticides (Lacey and Georgis, 2012). However, contrarily to Bt, they currently only play a minor role on the market for microbial insecticides. Commercial products for pest control are based on formulations of entomopathogenic nematodes of the genera *Heterorhabditis* and *Steinernema* with select strains of *Photorhabdus* and *Xenorhabdus* (Ehlers, 2001). Preparations of *Heterorhabditis* and *Steinernema *vectoring the entomopathogenic bacteria have been applied with varying success to control larval forms of some of the most notorious soil pest insects, including the black cutworm *Agrotis ipsilon* of the order Lepidoptera, *Diabrotica* spp. and *Diaprepes* sp. and *Otiorhynchus* sp. root weevils of the Coleoptera, and the cabbage root fly *Delia radicum* and fungus gnats (*Sciaridae*) of the Diptera (Denno et al., 2008; Lacey and Shapiro-Ilan, 2008; Degenhardt et al., 2009; Toepfer et al., 2010; Campos-Herrera et al., 2012 ). The two nematodes have also been used in combination with the entomopathogenic fungus *Metarhizium *and Bt maize to improve root protection from damage caused by *Diabrotica* spp. (Petzold-Maxwell et al., 2013).

*Photorhabdus* and *Xenorhabdus *are fascinating entomopathogenic bacteria and they have been studied extensively for their insect pathogenicity and mutualistic interaction with nematodes, as well as for their production of an array of protein toxins and toxic secondary metabolites with insecticidal potential (ffrench-Constant et al., 2007; Herbert and Goodrich-Blair, 2007; Bode, 2009; Waterfield et al., 2009; Nielsen-LeRoux et al., 2012). They provide a rich source of novel insecticidal toxins for crop protection, as it will be exemplified later in this review. There have been efforts to isolate new strains of these entomopathogens to mine for novel antimicrobial and insecticidal compounds (Thanwisai et al., 2012), and to create insect-resistant plants using toxins from *Photorhabdus luminescens* (Liu et al., 2003). In contrast to Bt, which relies on the oral route of infection in order to kill the insect host, *Photorhabdus* and *Xenorhabdus* species are “delivered” directly into the insect hemocoel by their nematode vectors, which then invade the insects either via penetration of the cuticle or through natural openings. As a result, this infection strategy makes the bacteria dependent on their nematode symbiont, which in turn makes applications of these microorganisms for insect pest management in the soil much more complex than it would be in the case of a free-living, entomopathogenic rhizobacterium.

**FIGURE 1:**
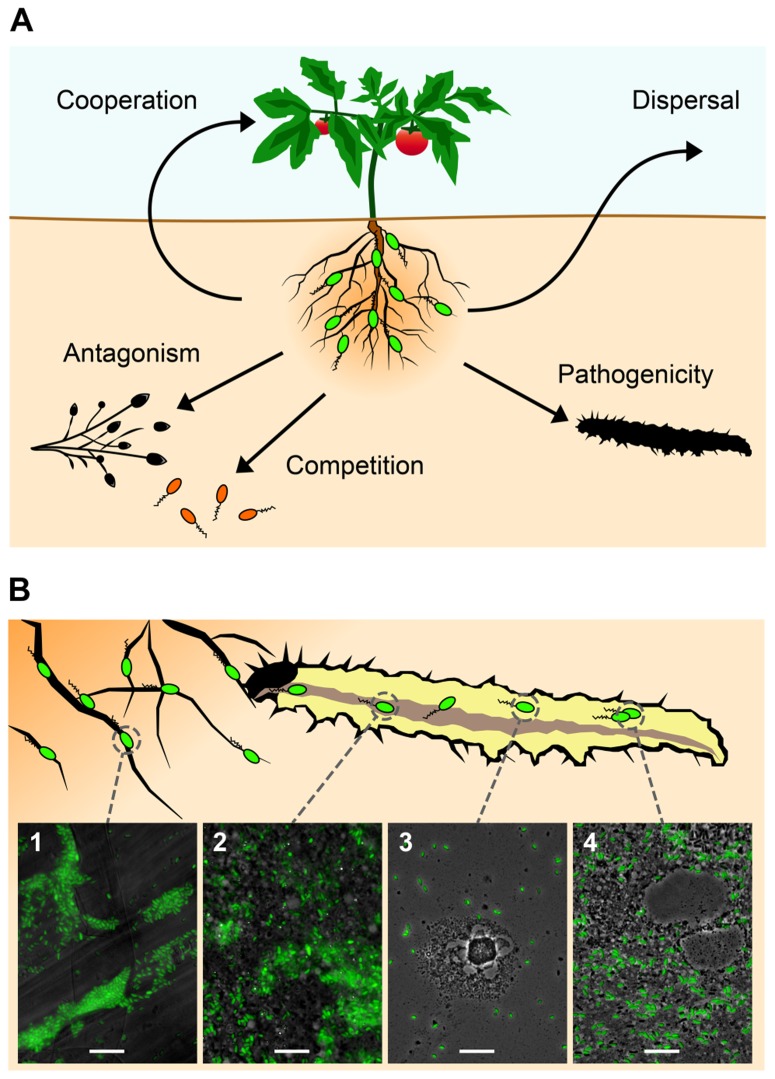
**Certain plant root-associated *Pseudomonas* bacteria exhibit insect pathogenicity as an additional trait to the well-studied biocontrol activity against phytopathogens (see text for more details).(A)** The most important interactions of these plant-beneficial pseudomonads (in green) include cooperation with the plant host (growth promotion and induction of systemic resistance) and competition with and antagonism of soil-borne phytopathogens. In addition, they show insecticidal activity and can use insects as vectors for dispersal. **(B)** Certain strains of *Pseudomonas protegens* and *Pseudomonas chlororaphis* are capable of infecting and efficiently killing insect larvae after oral uptake. *P. protegens* strain CHA0 (here tagged with GFP for microscopical visualization) typically forms microcolonies on roots (1) of various plant species (here tomato). Following ingestion by herbivorous insects, the entomopathogenic *P. protegens *strain is able to colonize the midgut (2) of pest insect larvae (here the large cabbage white *Pieris brassicae*), possibly by competing with the intestinal microbiota. By a so far unknown mechanism CHA0 cells then cross the intestinal epithelial barrier and invade the hemocoel within less than 1 day after oral infection (3). Once in this body compartment, the bacteria proliferate, resist uptake and elimination by hemocytes and cause disease (4). Bars represent 10 μm.

In this review, we present the very first detailed overview about insect interaction and insecticidal activity in pseudomonads, and we illustrate that certain root-associated bacteria of the genus *Pseudomonas* could constitute a promising alternative to the above-mentioned two groups of commercialized entomopathogens, in particular when addressing the notorious problem of soil-dwelling pests. As described in more detail below, these well-known rhizobacteria are capable of protecting plant roots against fungal and oomycete pathogens and simultaneously show potent oral insecticidal activity (**Figure 1A**). Some of these bacterial strains are already successfully used as antifungal biocontrol agents in agriculture (Berg, 2009). Therefore, these root-associated bacteria could be exploited for the development of novel microbial products which would protect plant roots simultaneously against phytopathogens and herbivorous insects and could become an important element of IPM.

## INTERACTION OF BENEFICIAL PSEUDOMONADS WITH PLANTS AND PHYTOPATHOGENS: COOPERATION, COMPETITION, AND ANTAGONISM

The genus *Pseudomonas *makes up a remarkably ubiquitous and diverse group of microorganisms. These Gram-negative bacteria are highly adaptive and can use a wide variety of compounds as an energy source, and as a result, there is practically no place on earth where they cannot be found (Wu et al., 2010; Silby et al., 2011). The environmental niches that they colonize range from oil-spilled seawater (Viggor et al., 2013) to soil (Weller et al., 2002), plant surfaces (Hirano and Upper, 2000; Loper et al., 2012), and insect guts (Vodovar et al., 2005). Some of them live a life as saprophytes, while some are plant pathogens or opportunistic human pathogens, and yet others entertain commensal or almost mutualistic relationships with plants. The latter are in most cases root-colonizing members of the *Pseudomonas fluorescens* group according to Mulet et al. (2010, 2012b), and include amongst others the species *Pseudomonas fluorescens*, *Pseudomonas protegens*, and *Pseudomonas chlororaphis*. Among them, plant-beneficial pseudomonads are well-known for their multiple skills that enable them to not only survive and compete in the rhizosphere, which is an ecological hot spot attracting many different kinds of organisms, but also to undergo intimate interactions with the plant itself (**Figure 1A;** Lugtenberg and Kamilova, 2009; Hol et al., 2013). To this effect, the root-colonizing pseudomonads first became renowned thanks to the ability of some strains to protect plants against the attack by some of the most notorious soil-borne fungal and oomycete pathogens, including *Gaeumannomyces*, *Thielaviopsis*, *Rhizoctonia, Fusarium oxysporum,* and *Pythium *sp. (**Table 1;** Cook et al., 1995; Haas and Défago, 2005; Mercado-Blanco and Bakker, 2007).

**Table 1:** Prominent root-associated *Pseudomonas fluorescens* group strains with biocontrol activity against plant diseases and effectors contributing to pathogen suppression.

Strain^a^	Target soil-borne // leaf pathogens^b^	Pathogen suppression mechanisms^c^	Effectors (antibiotics/biosurfactants // siderophores)^d^	Reference^e^
***Pseudomonas protegens***
****CHA0	*Thielaviopsis, Pythium, Gaeumannomyces, Rhizoctonia, Fusarium* // *Hyaloperonospora*, TNV	Antibiosis, ISR	DAPG, pyrrolnitrin, pyoluteorin, HCN/orfamide // pyoverdine, enantiopyochelin	Haas and Keel (2003), Haas and Défago (2005), Youard et al. (2011)
Pf-5	*Pythium, Rhizoctonia, Drechslera, Sclerotinia* // *Pst*	Antibiosis, ISR	DAPG, pyrrolnitrin, pyoluteorin, HCN, rhizoxins/orfamide // pyoverdine, enantiopyochelin	Gross and Loper, 2009, Loper et al., 2012, Weller et al. (2012)
***Pseudomonas chlororaphis***
30-84	*Gaeumannomyces*	Antibiosis	Phenazines, pyrrolnitrin, HCN // pyoverdine	Pierson and Pierson (2010);, Loper et al. (2012)
O6	*// Phytophthora, Corynespora, Pectobacterium*	Antibiosis, ISR	Phenazines, pyrrolnitrin, HCN // pyoverdine	De Vleesschauer and Hífte (2009);, Park et al. (2011), Loper et al. (2012)
PCL1391	*Fusarium*	Antibiosis	Phenazines, HCN // pyoverdine	Chin-A-Woeng et al. (2001);, Ruffner (2013)
***Pseudomonas fluorescens***
2-79	*Gaeumannomyces*	Antibiosis	Phenazine // pyoverdine	Weller (2007);, Mavrodi et al. (2010)
DR54	*Pythium, Rhizoctonia*	Antibiosis	/Viscosinamide // pyoverdine	Nielsen and Sørensen (2003)
F113	*Pythium, Fusarium, Pectobacterium*	Antibiosis	DAPG, HCN // pyoverdine	Redondo-Nieto et al. (2013)
Pf29A	*Gaeumannomyces*	Alteration of fungal pathogenesis	ND	Daval et al. (2011);, Marchi et al. (2013)
Q2-87	*Gaeumannomyces* // *Pst*	Antibiosis, ISR	DAPG, HCN // pyoverdine	Loper et al. (2012);, Weller et al. (2012)
SBW25	*Pythium*	ND	/Viscosin // pyoverdine	Loper et al. (2012);, Trippe et al. (2013)
SS101	*Pythium* // *Phytophthora, Pst*	ISR	/Massetolide // pyoverdine	Loper et al. (2012);, van de Mortel et al. (2012)
WCS374	*Fusarium* // *Magnaporthe, Pst*	ISR	// Pyoverdine, pseudomonine	Bakker et al. (2007);, De Vleesschauer and Hífte (2009)
WCS417	*Fusarium* // *Alternaria, Hyaloperonospora*, *Botrytis, Pst*	ISR	ND	Bakker et al. (2007);, Van der Ent et al.(2008)

a Strains belonging to the *P. fluorescens* group according to Mulet et al. (2010), 2012b.

b Pst, *Pseudomonas syringae* pv. *tomato*; TNV, tobacco necrosis virus.

c ISR, induced systemic resistance. ND, not determined.

d Major effectors with antimicrobial, biosurfactant, metal-chelating, and/or plant defense-inducing properties produced by the respective strain. DAPG, 2,4-diacetylphloroglucinol; HCN, hydrogen cyanide.

e References from which further information on the strains can be accessed.

The mechanisms by which pseudomonads suppress plant diseases have been studied for many years. These bacteria are excellent root colonizers and compete effectively with pathogens for rhizosphere niches and macro- and micronutrients (Mercado-Blanco and Bakker, 2007; Lugtenberg and Kamilova, 2009). Notably, pseudomonads produce high affinity iron chelators (so-called siderophores such as pyoverdines and pyochelins) by which they sequester iron, which is in limited supply in soil, and render it unavailable for the pathogens (**Table 1;** Keel et al., 1989; Loper and Buyer, 1991; Cornelis, 2010; Youard et al., 2011). Probably the most potent mechanism by which pseudomonads can suppress soil-borne pathogens is antibiosis (Haas and Keel, 2003). Many disease-suppressive strains produce one, two, or even an entire cocktail of secondary metabolites with potent antifungal activity by which they can ward off plant pathogens. Phenazines, 2,4-diacetylphloroglucinol (DAPG), pyoluteorin, pyrrolnitrin, hydrogen cyanide (HCN), and cyclic lipopeptides are metabolites with a documented role in disease suppression (**Table 1;** Haas and Keel, 2003; de Werra et al., 2008; Gross and Loper, 2009; Mentel et al., 2009; Raaijmakers et al., 2010; Rochat et al., 2010; Jousset et al., 2011). The pseudomonads use several of these compounds also for self-defense against predatory protozoa and nematodes (Bjørnlund et al., 2009; Jousset et al., 2009; Raaijmakers and Mazzola, 2012). Most remarkably, root-inhabiting pseudomonads producing DAPG, phenazines, or cyclic lipopeptides are key components of soils that are naturally suppressive to specific soil-borne diseases such as take-all of wheat, black root of tobacco, and *Rhizoctonia* root rot of sugar beet (Weller et al., 2002; Haas and Défago, 2005; Mazurier et al., 2009; Mendes et al., 2011; Almario et al., 2013).

Several root-associated *Pseudomonas *strains are able to reduce plant diseases not only by directly antagonizing pathogens but also indirectly by activating plant defenses (**Table 1**). The beneficial effects of induced systemic resistance (ISR) triggered by root-colonizing pseudomonads in mono- and dicotyledonous plants against plant pests caused by fungal, oomycete, bacterial and viral pathogens, and also by herbivorous insects are extensively documented (Maurhofer et al., 1994; Bakker et al., 2007; De Vleesschauer and Höfte, 2009; van de Mortel et al., 2012; Zamioudis and Pieterse, 2012; Balmer et al., 2013). Nevertheless, pseudomonads sometimes can also negatively interfere with plant defenses against insects or with the attraction of parasitoids of leaf-feeding insects (Pineda et al., 2012, 2013). A number of bacterial determinants eliciting ISR have been identified, including iron chelators such as pyoverdines and pyochelins, and antimicrobials such as DAPG, phenazines, and lipopeptides (**Table 1;** Bakker et al., 2007; De Vleesschauer and Höfte, 2009). Most pseudomonads that are capable of inducing systemic resistance do this by priming plants in a way which leads to an accelerated, mostly jasmonate-signaling dependent response upon pathogen or insect attack (Prime-A-Plant Group, 2006; Bakker et al., 2007; De Vleesschauer and Höfte, 2009).

There are two main strategies by which we can exploit these pseudomonads with their astonishing repertoire of plant-beneficial activities for improving crop performance and crop health. The first is to adapt cropping systems in a way that attracts the beneficial rhizobacteria, fosters their populations, and stimulates their activity (Janvier et al., 2007; Berendsen et al., 2012). This may be achieved in numerous ways, e.g., by adapting tillage or crop rotation practices, by soil amendments such as quality composts or by the use of inter- or covercrops (Mazzola, 2004; Janvier et al., 2007). The second strategy is to apply *Pseudomonas*-based biopesticides either as a seed treatment, soil drench, or foliar spray. Several products based on plant-beneficial pseudomonads for use in integrated biological control have been commercialized mainly for the US market, including AtEze (*P. chlororaphis*) with activity against *Pythium*, *Rhizoctonia*, and *Fusarium* root diseases of vegetables and ornamentals in greenhouses, BlightBan A506 (*P. fluorescens*) used against fire blight on apple and pear, and Bio-Save 10 LP/11 LP (*Pseudomonas syringae*) used for the control of post-harvest diseases of fruits and potato (Fravel, 2005). In several European countries, two formulations based on *P. chlororaphis*, i.e., Cedomon and Cerall, are sold as a seed treatment against seed-borne diseases of cereals (Mark et al., 2006) and the *Pseudomonas*-based product Proradix (Buddrus-Schiemann et al., 2010) was recently placed on the market for use as a potato tuber treatment against diseases caused by *Rhizoctonia*, *Phytophthora, Streptomyces*, and *Erwinia*. Considerations for the selection, production, delivery, field testing, and registration of *Pseudomonas* and other biocontrol agents for commercial purposes have been reviewed elsewhere (Walsh et al., 2001; Fravel, 2005; Mark et al., 2006; Berg, 2009; Höfte and Altier, 2010).

## INSECTICIDAL ACTIVITY IN PLANT-BENEFICIAL *P. fluorescens* GROUP BACTERIA: OCCURRENCE AND MOLECULAR BASIS

Until very recently, insecticidal activities in the *P. fluorescens* group had only been sparsely documented (**Table 2**). Notably, strains of *P. fluorescens *were reported to exhibit insecticidal activity toward agricultural pest insects such as aphids (Hashimoto, 2002), phytophagous ladybird beetles (Otsu et al., 2004), and termites (Devi and Kothamasi, 2009). In the same vein, a bioformulation of a combination of two *P. fluorescens* strains was demonstrated to simultaneously reduce the incidence of a herbivorous insect (the rice leafroller *Cnaphalocrocis medinalis*) and a phytopathogenic fungus (*Rhizoctonia solani*) in rice under greenhouse and field conditions (Commare et al., 2002; Karthiba et al., 2010). Furthermore, a number of *P. fluorescens* strains were found to be capable of either killing the common fruit fly *Drosophila melanogaster *or of causing morphological defects to the widely used laboratory insect (de Lima Pimenta et al., 2003; Olcott et al., 2010). Although in some cases protein extracts (Prabakaran et al., 2002) or metabolites of *P. fluorescens *group strains, such as HCN (Devi and Kothamasi, 2009) and the lipopeptides viscosin (Hashimoto, 2002) and orfamide (Jang et al., 2013), were shown to have insecticidal properties, the molecular basis and regulation of the insecticidal activity in these bacteria remains obscure.

**Table 2:** Insecticidal activity in *Pseudomonas* species and currently known effectors and regulatory mechanisms involved in insect virulence.

Bacterial strain^a^	Target insect	Application of bacteria / bacterial product^b^	Effector / regulatory mechanism involved in insect virulence^c^	Reference
***Pseudomonas protegens***
**CHA0**	*Galleria mellonella, Manduca sexta*	Injection	Fit toxin (similar to Mcf toxin of *Photorhabdus*)	Péchy-Tarr et al. (2008), 2013
	*Spodoptera littoralis*	Feeding (D, L)**	Fit toxin, GacA (global regulator of virulence and biocontrol)	Ruffner et al. (2013)
	*Heliothis virescens, Plutella xylostella*	Feeding (L)**	ND	Ruffner et al. (2013)
	*Odontotermes obesus*	Contact (live cells)**	HCN (biocide)	Devi and Kothamasi (2009)
Pf-5	*G. mellonella, M. sexta*	Injection**	Fit toxin	Péchy-Tarr et al. (2008)
	*Drosophila melanogaster*	Feeding (D)**	GacA	Olcott et al. (2010)
F6	*Myzus persicae*	Contact (purified metabolite)**	Orfamide (biosurfactant)	Jang et al. (2013)
***Pseudomonas chlororaphis***
30-84	*G. mellonella*	Injection**	ND	Ruffner (2013)
PCL1391	*S. littoralis*	Feeding (D, L)	Fit toxin	Ruffner et al. (2013)
	*H. virescens, P. xylostella*	Feeding (L)	ND	Ruffner et al. (2013)
ST-1	*Bombyx mori*	Injection**	ND	Tao et al. (2011)
***Pseudomonas fluorescens***
AH1, FP7 and Pf1	*Cnaphalocrocis medinalis*	Feeding (L)**	ND	Commare et al. (2002);, Karthiba et al.(2010)
HS870031	*Myzus persicae, Aphis gossypii, Aulacorthum solani*	Contact (purified metabolite)**	Viscosin (biosurfactant)	Hashimoto (2002)
KPM-018P	*Epilachna vigintioctopunctata*	Feeding (oral injection, L)	ND	Otsu et al. (2004)
MF37	*D. melanogaster*	Pricking	Adherence factors (LPS, OMP)	de Lima Pimenta et al. (2003)
NN, Biotype C	*Apis mellifera*	Feeding (D)**	ND	Horn and Eberspächer (1976)
NN	*Formica paralugubris*	Contact (live cells)	ND	Chapuisat et al. (2007)
SBW25	*D. melanogaster*	Feeding (D)	ND	Olcott et al. (2010)
***Pseudomonas taiwanensis***
TKU015	*D. melanogaster*	Feeding (purified toxin)**	TccC-like toxin (similar to *Photorhabdus* toxin complex component TccC)	Liu et al. (2010)
***Pseudomonas sp.***
EP-3	*M. persicae*	Contact (purified metabolite)**	Rhamnolipid (biosurfactant)	Kim et al. (2011)
ICTB-745	*Rhyzopertha dominica*	Contact (purified metabolites)**	Rhamnolipids, PCA (antibiotic)	Kamal et al. (2012)
***Pseudomonas entomophila***
L48	*D. melanogaster*	Feeding (D)	Monalysin (pore-forming toxin), AprA (metallo-protease), GacA, Pvf (signaling system), AlgR (regulator)	Vodovar et al. (2005), 2006, Liehl et al. (2006);, Vallet-Gely et al. (2010b), Opota et al. (2011)
	*G. mellonella*	Force feeding of live cells	GacA	Fedhila et al. (2010)
***Pseudomonas syringae***
B728a	*Acyrthosiphon pisum*	Feeding (D, L)	FliL (flagellum formation and motility)	Stavrinides et al. (2009)
CHA	*D. melanogaster*	Pricking	T3SS and effectors (ExoS)	Fauvarque et al. (2002), Avet-Rochex et al. (2005)
PA14	*G. mellonella*	Injection	T3SS and effectors (ExoT, ExoU)	Miyata et al. (2003)
	*D. melanogaster*	Feeding (D)**	Quorum sensing (RhlR)	Limmer et al. (2011)
PAO1	*D. melanogaster*	Injection**	HCN	Broderick et al. (2008)	
	*B. mori*	Injection**	Superoxide dismutase (SodM, SodB), exotoxin A, GacA	Chieda et al. (2005), 2011, Iiyama et al. (2007)
	*B. mori*	Midgut injection**	ExoS, pyoverdine (iron chelator)	Okuda et al. (2010)
	*D. melanogaster*	Feeding (D)**	Quorum sensing (QscR), stringent response (ppGpp), control of biofilm formation	Chugani et al. (2001);, Mulcahy et al. (2011), Vogt et al. (2011);, de Bentzmann et al. (2012)
	*Pieris rapae*	Feeding (D)**	Quorum sensing (LasI, RhlI)	Borlee et al. (2008)
NN	*Melanoplus bivittatus*	Injection, Feeding (L)	ND	Bucher and Stephens (1957), Stephens (1958)

a NN, not named.

b Injection, bacterial cell suspension injected into the hemocoel if not mentioned otherwise. Feeding, oral administration of a bacterial cell suspension with artificial diet (D) or applied to plant leaves (L); Contact, bacterial cells or products sprayed on or put otherwise in contact with insect surface.

c ND, not determined; HCN, hydrogen cyanide; LPS, lipopolysaccharide; OMP, outer membrane protein; PCA, phenazine-1-carboxylic acid; T3SS, type III secretion system.

The genome sequencing of the root-colonizing biocontrol agent *P. fluorescens *strain Pf-5 (now called *P. protegens* Pf-5; Ramette et al., 2011) published by Paulsen et al. (2005) and of the closely related *P. fluorescens* strain CHA0 (recently renamed *P. protegens* CHA0; NCBI Database Bioproject PRJNA78307) and their analysis revealed astonishing results which opened a new door to future studies on plant-associated pseudomonads. After more than twenty years of research on the biocontrol properties of *P. fluorescens* group strains it came as a surprise that some of these bacteria do not only harbor numerous genes for the biosynthesis of antifungal metabolites, including DAPG, pyoluteorin, HCN, and pyrrolnitrin (see **Table 1**), in their genomes, but also possess a gene which codes for a protein that is similar to the potent insect toxin Mcf1 of the entomopathogen *P. luminescens *(Péchy-Tarr et al., 2008).

Mcf1 was discovered in a screening of a *P. luminescens* W14 cosmid library aiming at the identification of new insecticidal proteins and metabolites in this entomopathogenic bacterium (Daborn et al., 2002). A single gene which was called *makes caterpillars floppy* (*mcf*) made the *Escherichia coli* cells expressing it capable of surviving within and killing larvae of the tobacco hornworm *Manduca sexta* upon injection into the hemocoel. When expressed heterologously in *E. coli*, Mcf1 was shown to cause hemocytes and midgut epithelial cells to undergo programmed cell death. The disintegration of the midgut caused by Mcf1 was proposed to contribute to the “floppy” phenotype of insects infected with *P. luminescens*, thereby giving the name to the newly discovered toxin. The pro-apoptotic action of Mcf1 was attributed to the predicted Bcl2-homology 3-like (BH3-like) domain at the N-terminus of the protein. The BH3 domain is a well-studied and important peptide motif of proteins making up part of the pro-apoptotic signal-transduction cascades in animal cells (Cory and Adams, 2002). Mcf1 has been shown to also trigger apoptosis in mammalian cells and the N-terminal part of the toxin containing the BH3-like domain was sufficient for the observed toxicity (Dowling et al., 2004). The potent insect toxin seems to hijack the apoptosis cascades of the cells of the innate immune system and thereby to contribute to the immune suppressive activity of *P. luminescens*.

An exciting feature of the *mcf1*-related gene of *P. fluorescens* group strains Pf-5 and CHA0 is that, in contrast to *mcf1*, it is part of an eight-gene cluster (Péchy-Tarr et al., 2008). The cluster was termed *fit* for *P. fluorescens *insecticidal toxin. The gene *fitD,* which codes for the actual insect toxin with a molecular weight of 327 kDa, is flanked by four genes (*fitABC-E*) predicted to encode a type I secretion system and three genes (*fitFGH*) coding for regulatory proteins. The toxin gene is co-transcribed with the genes encoding the proteins for the putative secretion system, thereby suggesting that the toxin may be transported across the bacterial cell wall via this type I secretion system (Péchy-Tarr et al., 2013). While the transport of the Fit toxin still remains to be investigated, the roles and importance of the individual regulatory proteins of the Fit cluster have been elucidated and are described in more detail below. Because the putative BH3-like domain of Mcf1 is also conserved in the Fit toxin, it is imaginable that FitD induces apoptosis in insect cells as well.

So far the Fit toxin gene has been detected in the genomes of only a narrow group of plant-associated pseudomonads, namely in isolates of *P. protegens* and *P. chlororaphis *(**Table 2;** Loper et al., 2012; Ruffner et al., 2013; Shen et al., 2013). Strains of these two bacterial species generally showed a high toxicity toward larvae of lepidopteran insects. The *P. protegens *strains CHA0 and Pf-5 were lethal to larvae of *M. sexta* and the greater wax moth *Galleria mellonella* upon injection of very low doses into the hemocoel of these insects (Péchy-Tarr et al., 2008). The Fit toxin thereby significantly contributed to the insecticidal activity of these microorganisms. Furthermore, as with Mcf1, heterologous expression of the Fit toxin in *E. coli* resulted in the capacity of the bacterium to kill the insect host upon injection.

*P. protegens* strain CHA0 and *P. chlororaphis* strain PCL1391 were later also shown to display potent oral insecticidal activity in feeding assays with artificial diet or leaves treated with the bacteria (**Table 2;** Ruffner et al., 2013). When bacterial suspensions containing low cell concentrations were sprayed on plant leaves, both strains efficiently killed larvae of several agriculturally important lepidopteran pest insects, notably the African cotton leafworm *Spodoptera littoralis*, the tobacco budworm *Heliothis virescens*, and the diamondback moth *Plutella xylostella* that fed on the leaves. The Fit toxin was found to substantially contribute to the oral insecticidal activity of the two model strains. In contrast, a related but naturally Fit-deficient *P. fluorescens* group strain displayed almost no oral toxicity in the same assay (Ruffner et al., 2013). Thus the presence of the Fit toxin gene in plant-colonizing pseudomonads seems to correlate well with high toxicity of these strains toward insects. This and observations with additional strains suggest that the gene could potentially be used as a suitable molecular marker for insecticidal activity in fluorescent pseudomonads (Ruffner et al., 2009; Ruffner, 2013). In addition to the Fit toxin, traits regulated by the GacS/GacA two-component system, which is known to control pathogenic and beneficial activities in pseudomonads (Haas and Keel, 2003; Lapouge et al., 2008), contribute significantly to the oral insecticidal activity of *P. protegens* CHA0 (Ruffner et al., 2013). Additional toxicity assays suggest specificity in the insecticidal spectrum of *P. protegens* CHA0. In particular, during a quest for potential side effects of the pseudomonad toward beneficial insects, the Fit toxin producers were found to exhibit no oral toxicity toward an ecologically and economically important pollinator, the large earth bumblebee *Bombus terrestris* (Ruffner, 2013).

The potential of these plant root-associated pseudomonads as entomopathogenic microorganisms can be demonstrated impressively by feeding Chinese cabbage leaves containing drops of a suspension of GFP-tagged *P. protegens *CHA0 to larvae of the large cabbage white *Pieris brassicae*. The bacteria seem to be capable of colonizing the insect gut and subsequently translocating into the hemocoel by so far unknown means, where they replicate and cause disease (**Figure 1B**). The invasion of the insect blood system within a short time period of less than 1 day after oral uptake of the microorganisms strongly suggests that these bacteria should be considered as true insect pathogens.

Because of the genetic organization of the *fit* cluster and the fact that the regulation of virulence in entomopathogenic bacteria has been addressed only to a limited extent at the molecular level before, it was particularly intriguing to study of the expression of the Fit toxin and its regulation. To this end the Fit toxin gene of *P. protegens* CHA0 was replaced at its native locus by a gene fusion of *fitD* to *mcherry *(coding for a red fluorescent protein), which allowed the direct *in situ* visualization and quantification of insect toxin production at wild-type level by monitoring and measuring the red fluorescence emitted by single cells with an epifluorescence microscope (Péchy-Tarr et al., 2013). The reporter strain expressed the Fit toxin only during an infection of insect larvae, but not when growing on plant roots or in common laboratory media. This indicated that the expression of the insect toxin is activated in a host-dependent manner and is tightly controlled in these bacteria. According to the current molecular model about the roles of the individual local regulators, all three proteins appear to be crucial for the observed tight regulation of toxin production in *P. protegens* CHA0 (Péchy-Tarr et al., 2013). The sensor histidine kinase-response regulator hybrid FitF is thought to perceive a yet unknown signal and to inactivate the repressor protein FitH by phosphorylation upon infection of an insect. This most probably releases the activator FitG, a member of the family of LysR-type transcriptional regulators, from the inhibition by FitH and leads to the activation of toxin expression. The observed production of the Fit toxin in insects and the molecular study of the three local regulators indicate that *P. protegens* is capable of detecting the insect host and of actively inducing the production of the Fit toxin during an infection of insect larvae.

However, the deletion of the Fit toxin gene in the chromosomes of *P. protegens* or *P. chlororaphis* strains is not sufficient to render them non-toxic to insects (Péchy-Tarr et al., 2008, 2013; Ruffner et al., 2013). This suggests that additional virulence factors are waiting to be discovered in these insecticidal pseudomonads. Candidate virulence factors that could play a role in insect pathogenicity in some of these strains are the so-called toxin complexes (Tc). Tc, which were first identified in *P. luminescens*, are large multimeric insecticidal protein complexes displayed on the surface of these bacteria (Bowen et al., 1998; ffrench-Constant et al., 2007). Although the exact mode of action of these orally active toxins is still not fully resolved, recent studies provide evidence that some Tc subunits function as a molecular syringe allowing membrane translocation of functional Tc components that induce actin clustering and death in target cells (Lang et al., 2010; Gatsogiannis et al., 2013). Tc components have also been investigated as alternatives to the Bt toxins for the development of transgenic crops (Liu et al., 2003). Tc-related gene clusters occur in many other bacteria that interact with insects, including *Xenorhabdus nematophila*, *Yersinia pestis*, *Yersinia entomophaga*, *Serratia entomophila*, and Bt (Hurst et al., 2000; Waterfield et al., 2001; Blackburn et al., 2011; Landsberg et al., 2011; Spinner et al., 2012). Remarkably, Tc-related genes can also be found in certain strains of *P. chlororaphis* and *P. fluorescens* (Loper et al., 2012) and their role in insect pathogenicity should thus be investigated in future studies. In pseudomonads, a role for a Tc-related gene so far has only been demonstrated for *tccC* from *Pseudomonas taiwanensis *of which the purified product caused substantial mortality when fed to larvae of *Drosophila* (Liu et al., 2010).

## MOLECULAR BASIS OF INSECT INTERACTION IN PROMINENT PATHOGENIC PSEUDOMONADS

Several observations suggest that natural interactions of pseudomonads with insects are most likely more widespread than recognized so far. First, members of the genus *Pseudomonas* make commonly part of microbial communities of various insect species. Indeed, using culture-dependent and -independent approaches, pseudomonads were identified as common inhabitants of the intestinal tract or otherwise associated with field-collected or laboratory-raised larvae, pupae, and adults of representatives of the major insect orders. Examples include *Anopheles*, *Aedes*, and *Culex* mosquitoes, the *Drosophila* fruit fly, and the Hessian fly *Mayetiola destructor *in the order Diptera (Corby-Harris et al., 2007; Bansal et al., 2011; Osei-Poku et al., 2012), *S. littoralis*, the cotton bollworm *Helicoverpa armigera*, and the gypsy moth *Lymantria dispar* in the Lepidoptera (Broderick et al., 2004; Tang et al., 2012), the wireworm *Limonius canus*, the forest cockchafer *Melolontha hippocastani*, and *Periplaneta* and *Blattella* cockroaches in the Coleoptera (Lacey et al., 2007; Saitou et al., 2009; Arias-Cordero et al., 2012), *Camponotus* ants and several bee species in the Hymenoptera (Mohr and Tebbe, 2006; Li et al., 2012), and the leafhopper *Homalodisca vitripennis* and several aphids in the Hemiptera (Hashimoto, 2002; Lacava et al., 2007). Many of these insects feed on roots or aboveground parts of plants or spend a part of their life cycle in aquatic habitats, i.e., in environments that are typically colonized by pseudomonads. It is therefore likely that pseudomonads are commonly acquired by insects via ingestion or contact. These highly versatile bacteria then may be very well-adapted to live inside or otherwise associated with their arthropod host, exploiting it as a shelter, vector, or food source.

Second, the genomes of many *Pseudomonas* strains contain genetic loci with predicted function in insect interaction and insect toxicity. These loci are related to genes encoding known insect virulence determinants in the entomopathogens *Photorhabdu*s and *Xenorhabdus*, namely the Mcf toxins, the Tc toxin complexes, the XaxAB cytolysin, and several lytic enzymes (ffrench-Constant et al., 2007; Vigneux et al., 2007; Lindeberg et al., 2008; Stavrinides et al., 2009; Silby et al., 2011; Loper et al., 2012). To date, the function of most of these loci in pseudomonads remains nebulous. A clear role in insect toxicity so far has only been established for the Mcf homolog Fit (see above).

Third, following oral infection several *Pseudomonas* species are capable not only of colonizing insects but also of exhibiting significant pathogenicity toward insects. Besides the above-described plant-beneficial *P. protegens* and *P. chlororaphis* of the *P. fluorescens* group (Mulet et al., 2012b), currently only three pathogenic species are known to be capable of efficiently killing insects, (i) the entomopathogen *Pseudomonas entomophila*, (ii) the opportunistic human pathogen *Pseudomonas aeruginosa*, and (iii) the plant pathogen *P. syringae* (**Table 2**). As detailed below, studies of the interactions of the three pathogens with insect hosts have significantly advanced our understanding of the molecular mechanisms involved in bacterial invasion of insects, escape from the insect immune response, gut and hemocoel colonization, and insect toxicity. They have also provided first insights into the ecology of vectoring of pseudomonads by insects. Studies on these pathogens can thus provide a valuable source of inspiration for future work on interactions of plant-beneficial pseudomonads with insects.

The entomopathogen *P. entomophila* is a bacterium that naturally infects *Drosophila* and originally was isolated from a fruit fly in Guadeloupe. The species which affiliates with the *Pseudomonas putida* phylogenetic group (Loper et al., 2012; Mulet et al., 2012a,b) is also pathogenic toward lepidopteran insects (Vallet-Gely et al., 2008; Fedhila et al., 2010). Following oral infection, this bacterium is capable of persisting in the gut of *Drosophila*, inducing local and systemic immune responses and, at high doses, of killing the insect, and thus constitutes an exciting model for studies into virulence and host immune defense mechanisms (Vodovar et al., 2005; Vallet-Gely et al., 2008, 2010b). *P. entomophila* virulence is multifactorial and depends on the GacS/GacA two-component system (Vodovar et al., 2005; Liehl et al., 2006). A second global regulatory system involving a yet unidentified signal molecule synthesized by the Pvf proteins contributes to control of *P. entomophila* virulence and immune response induction independently of GacS/GacA (Vallet-Gely et al., 2010b). Two important virulence factors have been identified in the entomopathogen. One is the Gac controlled metalloprotease AprA which counteracts the local immune response in the *Drosophila* gut via degradation of antimicrobial peptides (AMP) produced by the insect (Liehl et al., 2006). The other is a Gac and Pvf controlled pore-forming protein toxin termed Monalysin which contributes to the massive damage to the fly gut caused by *P. entomophila* in a mechanism involving suppression of immune and repair programs in the intestinal tract (Opota et al., 2011; Chakrabarti et al., 2012). However, both AprA- and Monalysin-deficient mutants (but not *gacA* mutants) retain some degree of insect toxicity pointing to the existence of additional virulence factors. The genomic sequence of *P. entomophila* reveals a number of loci that encode potential candidate virulence factors, e.g., Tc-related toxins, HCN, hemolysins, and lipopeptides (Vodovar et al., 2006), which await to be explored. One of these factors, a lipopeptide with a role in hemolytic activity, was recently determined not to be required for virulence in *Drosophila* (Vallet-Gely et al., 2010a).

*P. aeruginosa* is an opportunistic human pathogen (Gellatly and Hancock, 2013) and several strains are capable of infecting mammalian, invertebrate (nematodes and insects) and plant hosts, and these multihost interactions can be used to unravel conserved and variable virulence strategies of the bacterium (Mahajan-Miklos et al., 2000; Hendrickson et al., 2001; Kim et al., 2008). In general, the capability of *P. aeruginosa* to infect and kill insects was not used to investigate insect pathogenicity of the bacterium *per se* but rather to profit of convenient infection models for exploring the molecular basis of virulence of the human pathogen, even more as insects rely on innate defense mechanisms resembling those in mammalian hosts to fight microbial infections (Vallet-Gely et al., 2008). The entomopathogenic potential of the species was recognized already in reports dating back to the early last century (Bacot, 1911; Cameron, 1934; Bucher and Stephens, 1957; Angus, 1965). For instance, a *P. aeruginosa* isolate was reported to be responsible for a disease in laboratory rearings of grasshoppers (Bucher and Stephens, 1957). The authors demonstrated that the disease can be produced artificially by injecting the isolate into the hemocoel (LD_50_ of 10–20 cells per insect) or by feeding the insects with the bacterium (LD_50_ of about 10^4^ cells per insect). A follow-up study then provided evidence for the passage of small numbers of the *P. aeruginosa* isolate from the gut into the hemocoel (Stephens, 1958). A field experiment with the isolate to control grasshoppers was not successful (Baird, 1958; Angus, 1965).

A majority of recent studies on *P. aeruginosa* insect virulence rely on variations of two *Drosophila* infection models, i.e., the fly nicking and fly feeding models thought to reflect acute or chronic infections, respectively (Sibley et al., 2008; Apidianakis and Rahme, 2009). In the nicking model rapid killing within 1–2 days after pricking flies with a needle dipped into a bacterial culture is observed, whereas the feeding model allows to monitor an extended infection process of 1–2 weeks after ingestion of a high concentration of bacteria by the flies. Using these models, considerable strain variation in virulence of *P. aeruginosa* to *Drosophila* was observed (Lutter et al., 2012) coinciding with similar observations for *P. fluorescens* group bacteria (Olcott et al., 2010). The variations in the pathogenicity are likely to mirror differences in the genomic equipage with relevant virulence genes and in the regulation of these genes in the different strains. Virulence gene expression by *P. aeruginosa* in the *Drosophila* intestinal tract and as a consequence insect pathogenicity is also influenced by other microorganisms present in the gut (Sibley et al., 2008).

As for *P. entomophila*, *P. aeruginosa* virulence toward *Drosophila* is multifactorial. Following ingestion, *P. aeruginosa* is able to colonize various parts of the *Drosophila* intestinal tract, counteract the insect immune defense, cross the intestinal barrier, and proliferate in the hemolymph (Sibley et al., 2008; Limmer et al., 2011; Mulcahy et al., 2011). Global regulatory mechanisms involved in virulence control such as quorum sensing (QS) and the ppGpp-mediated stringent response are essential for the infection process (Chugani et al., 2001; Limmer et al., 2011; Vogt et al., 2011). The importance of QS signaling in the insect gut is highlighted in another feeding model involving the small cabbage white *Pieris rapae* in which interruption of QS signaling by mutation or by a chemical inhibitor reduced the virulence of *P. aeruginosa* (Borlee et al., 2008). In a recent study, *P. aeruginosa* was found to be capable of establishing a biofilm infection in the *Drosophila* crop following ingestion, thereby inducing an AMP immune response in the fly (Mulcahy et al., 2011). Remarkably, a mutant defective in biofilm formation had an improved capacity to cross the intestinal barrier and to disseminate into the hemolymph and was more virulent than the wild-type parent (Mulcahy et al., 2011). By contrast, hyperbiofilm strains were markedly less virulent to flies, an observation that was confirmed by another study (de Bentzmann et al., 2012) and is in accordance with the common association of biofilm formation with chronic infection in *P. aeruginosa *(Gellatly and Hancock, 2013) and other bacterial pathogens.

Multiple virulence traits of *P. aeruginosa* have a role in the acute infection model of *Drosophila* (Kim et al., 2008), including the capacity to suppress the insect’s AMP defense response (Apidianakis et al., 2005), HCN production (Broderick et al., 2008) and delivery of type III secretion system (T3SS) effectors (Fauvarque et al., 2002; Avet-Rochex et al., 2005). The variety of virulence factors contributing to acute infection is further highlighted by studies involving the silkworm *Bombyx mori *and the *Galleria* wax moth, two widely used lepidopteran model insects*.* The global regulator GacA (Chieda et al., 2005), the ADP-ribosylating exotoxin A (Chieda et al., 2011), and superoxide dismutases (Iiyama et al., 2007), but not pyocyanin (Chieda et al., 2008) contribute to injectable activity of *P. aeruginosa* in the silkworm model. Several T3SS effectors including ExoTare important for virulence in the *Galleria* injection model (Miyata et al., 2003). A T3SS effector (ExoS) is also required for virulence and translocation of *P. aeruginosa* from the midgut to the hemolymph in the *Bombyx* model (Okuda et al., 2010).

*P. syringae* is an important member of the phyllosphere bacterial community and well-known for its plant pathogenic, ice-nucleating, and epiphytic activities (Hirano and Upper, 2000). However, possible activities of *P. syringae* in interactions with insects so far have attracted only little attention. Interestingly, a recent study suggests that at least some *P. syringae* strains may exhibit significant insecticidal activity (Stavrinides et al., 2009). In the study, the bean pathogen *P. syringae* pv. *syringae* B728a was found to kill the pea aphid *Acyrthosiphon pisum* within less than 2 days when fed to the insect in artificial diet. By contrast, the tomato pathogen *P. syringae* pv. *tomato* DC3000 did not harm the aphid even though cell densities of the strain in infected insects raised to higher levels than those of strain B728a. In another study, *P. syringae* pv. *mori* did not survive in the intestinal tract of *Bombyx mori *larvae fed an artificial diet containing the phytopathogen (Watanabe et al., 1998). This may suggest that, as with strains of the *P. fluorescens* and *P. aeruginosa* groups, the capacity for potent insect pathogenicity is associated only with certain *P. syringae *pathovars or strains and as such depends on the genomic background of the respective strain. The molecular basis of aphid toxicity of *P. syringae* pv. *syringae* B728a is unclear. Similarly to many other *P. syringae* strains, the genome of B728a harbors sequences related to those encoding the *Photorhabdus* Tc toxin complexes (Lindeberg et al., 2008). However, these were not required for virulence of *P. syringae* B728a in the aphid model (Stavrinides et al., 2009).

The work of Stavrinides and colleagues puts forward another interesting aspect of *Pseudomonas*–insect associations. They show that following natural infection of pea aphids by *P. syringae* present on leaves, the bacteria multiply inside the insect host and then can be spread at high cell concentrations onto fresh leaf surfaces in the honeydew deposited by the aphids (Stavrinides et al., 2009; Nadarasah and Stavrinides, 2011). Only very few other reports provide experimental evidence for insect vectoring of pseudomonads. For instance, the root-associated bacterium *P. chlororaphis* was demonstrated to be transmitted between corn plants by the Southern corn rootworm *Diabrotica undecimpunctata howardi* feeding on roots colonized by the bacterium (Snyder et al., 1998). In other reports, *P. fluorescens* strains were found to persist in the gut of the Colorado potato beetle *Leptinotarsa decemlineata* fed with the bacteria in laboratory experiments or prior to overwintering in the field (Castrillo et al., 2000a,b). The ice-nucleation active bacteria markedly increased the supercooling point of the insects, leading the authors to speculate on a possibility for the biological control of the freeze-intolerant pest insects by reducing the survival of overwintering populations with a *Pseudomonas *treatment. Finally, insects may also be considered as potential vectors for the dispersal of biocontrol pseudomonads. This is documented by field experiments in which honeybees were successfully used to disseminate *P. fluorescens* strain A506, a biocontrol agent of fire blight and the active ingredient of the commercial product BlightBan A506, to pear and apple blossoms (Johnson et al., 1993). Together, all these studies illustrate that insects may not only constitute alternatives hosts for pseudomonads but also may serve as vectors and shelters for their survival and multiplication.

## POTENTIAL OF PSEUDOMONADS FOR THE CONTROL OF ROOT-FEEDING PEST INSECTS

As it was illustrated in earlier chapters of this review, natural isolates of *P. protegens* and *P. chlororaphis* possess multiple activities that are beneficial to the plant in terms of growth and protection against various pests. These include antagonism of soil-borne phytopathogens, plant growth promotion, induction of systemic resistance, and insect pathogenicity (**Figure 1**). It is therefore that these bacteria have a high potential as plant protection products. Because they can promote the growth of plants and protect plant roots against several pests simultaneously, *Pseudomonas*-based formulations may become products of high profit potential (Chandler et al., 2011). While plant root-associated pseudomonads have been successfully used for the formulation of commercial fungicides (Fravel, 2005; Berg, 2009), no insecticidal products with *Pseudomonas* strains as active ingredient currently exist on the market for biopesticides.

The way to a product based on root-associated pseudomonads for efficient plant protection against insects and phytopathogenic fungi obviously is not free of obstacles. Pseudomonads are known to be challenging microorganisms when it comes to formulation (Walsh et al., 2001). The survival of the bacteria during the manufacturing process and long-term storage is a critical issue. Furthermore, *Pseudomonas*-based products were reported to exhibit inconsistency under field conditions and they have raised some concerns of the general public about biosafety because this bacterial genus includes opportunistic human pathogens such as *P. aeruginosa*. As with every new biopesticide, the expensive and time-consuming registration procedure is a major hurdle for the successful application of a biocontrol agent (Bale et al., 2008). Nevertheless, the few strains of the *P. fluorescens* group which are approved in many countries for their use as fungicides in agriculture already went through the evaluation of environmental risks and the registration procedure. The products passed all tests on biosafety and efficacy, and these bacterial strains should therefore be studied for their effects on insects to possibly extend their application range in the future by modifying their formulations.

Novel *Pseudomonas* strains can also readily be isolated from various insect species. An obvious approach to discover strains with entomopathogenic potential could therefore be the isolation of pseudomonads from the respective target organism. During the selection of strains for a new plant protection product the efficacy of the bacterium as an insecticidal organism, the persistence and competition on plant roots, and the resistance during the formulation process should be considered (Walsh et al., 2001). Moreover, a detailed risk analysis needs to be performed to ensure that the bacterial strains have no deleterious effects on human health and on the environment. This requires amongst others more research on the molecular basis and regulation of insecticidal activity in these root-associated pseudomonads. The importance of such investigations is impressively illustrated by the above-described discovery of the sophisticated regulatory switch allowing *P. protegens* to launch Fit toxin expression specifically in an insect host while arresting production of the insecticidal factor on roots (Péchy-Tarr et al., 2013), thus procuring a natural containment mechanism for biocontrol. The collaboration of the scientific community with commercial companies may then be the key to the development and commercialization of new biopesticides based on entomopathogenic, root-associated *Pseudomonas* strains, just like the development of products such as Proradix, Cedomon, and Cerall already has demonstrated (Johnsson et al., 1998; Buddrus-Schiemann et al., 2010).

Microbial control agents are considered environmentally friendly and harmless to mammals, making them ideal components of IPM systems. Commercial insecticides based on entomopathogenic bacteria are mostly applied as inundative releases for short-term pest control when insect populations have already reached a certain threshold (Lacey et al., 2001). Many strains of the *P. fluorescens *group are well-adapted to the life on plant roots and show environmental persistence. These microbes are very competitive and aggressive root colonizers (Lugtenberg and Kamilova, 2009), and would thus ideally be applied as inoculations for long-term control before pest insects pose a problem to the particular plant population. As for the commercially available *Pseudomonas*-based biofungicides, it could be possible to apply entomopathogenic strains of *P. protegens* and *P. chlororaphis* as seed coatings for inoculative releases and thereby use these microorganisms in a preventative manner. Because plant-associated pseudomonads are already successfully used as biological fungicides in agriculture, insecticidal products for crop protection with entomopathogenic *Pseudomonas* bacteria as active ingredient could fit well into integrated systems. They would extend the existing toolbox for IPM and help to optimize the protection of plants against pest insects that feed on roots during at least a part of their life cycle and remain a challenging problem in many agricultural systems. As mentioned before, formulations with plant-beneficial pseudomonads possessing insecticidal activity could potentially be developed to provide products to the farmers that may permit long-term control of root-feeding insects and soil-borne phytopathogens simultaneously in an IPM framework. Future research should investigate the interaction of these pseudomonads with other IPM components. Combinations with other biocontrol agents such as entomopathogenic fungi or nematodes or further IPM tactics could show a synergistic effect on the suppression of plant pests (Lacey et al., 2001; Lacey and Shapiro-Ilan, 2008; Karthiba et al., 2010; Hol et al., 2013).

The analysis and comparison of whole genome sequences in order to find candidate genes or gene clusters contributing to the insecticidal activity is a powerful approach to discover novel virulence factors and to extend the knowledge about these bacteria. It is further important to learn from existing data on other entomopathogenic bacteria to get a better understanding of the relevant virulence factors and their regulation, the mechanisms of colonization and invasion, and other functions required for insect pathogenicity of the plant-beneficial *Pseudomonas* strains. This includes in particular research on *Photorhabdus/Xenorhabdus* and pathogenic *Pseudomonas* species, but also studies about less known bacteria capable of killing insects, e.g., the aphid-infecting plant pathogen *Dickeya dadantii* (Costechareyre et al., 2013), could be inspiring for future investigations. Moreover, it is fundamental to carry out future research related to the control of soil-dwelling pest insects by beneficial root-associated pseudomonads under (near) natural conditions. This implies investigations into the interactions of these biocontrol bacteria with the natural microbiota of the insect gut just as the assessment of the efficacy of killing of insects under field conditions. Such approaches may help lessen known problems of inconsistency of *Pseudomonas*-based products in the field from the beginning.

We think that the current knowledge about the insect pathogenicity of certain root-associated pseudomonads and the powerful tools that are available for further investigations into this exciting feature are promising and a motivation for the development and application of microbial pesticides based on well-selected strains of these bacteria for a better management of root-feeding pest insects in the near future.

## Conflict of Interest Statement

The authors declare that the research was conducted in the absence of any commercial or financial relationships that could be construed as a potential conflict of interest.
